# Post-pregnancy contraception

**DOI:** 10.1530/RAF-25-0180

**Published:** 2026-05-20

**Authors:** Michelle Cooper, Helena Kopp Kallner

**Affiliations:** ^1^ NHS Lothian & University of Edinburgh, Edinburgh, UK; ^2^ Danderyd Hospital & Karolinska Institutet, Stockholm, Sweden

**Keywords:** contraception, postpartum contraception, pregnancy

## Abstract

**Abstract:**

Good contraception care after pregnancy can remove barriers to accessing contraception and reduce future unintended pregnancy. While most methods can be started safely in the immediate post-pregnancy period, including long-acting reversible contraception (LARC) methods, there are some specific considerations that may influence their use post-pregnancy. This review summarises the current evidence and recommendations around post-pregnancy contraception following abortion, childbirth and early pregnancy loss (miscarriage, ectopic pregnancy and molar pregnancy), highlights practical considerations and identifies gaps for future research.

**Lay summary:**

Fertility can return very quickly following an abortion, childbirth or an early pregnancy loss. Without contraception, women can experience a pregnancy shortly after these episodes, which is often not what they intend. Having the opportunity to discuss future fertility plans and contraceptive options during these pregnancy episodes can empower women to start their chosen method of contraception early, reducing the need to attend appointments to do this later and the possibility of experiencing an unintended pregnancy.

## Introduction

Globally, it is estimated that at least 50% of all pregnancies are unintended at the time of conception (Bearak *et al.* 2018). While many unintended pregnancies result in abortion, where a pregnancy is continued, a lack of pregnancy planning has also been linked to poorer maternal and neonatal outcomes in many settings (Mohllajee *et al*. 2007). The post-pregnancy period is a high-risk time for future unintended pregnancy. Ovulation can return much sooner than individuals may expect and within a matter of days or weeks, depending on gestational length. While contraception is often discussed routinely in the context of abortion, international guidance increasingly recommends incorporating contraception discussion and provision across the continuum of pregnancy care, including after childbirth or pregnancy loss (Faculty of Sexual & Reproductive Healthcare 2017). Contraceptive counselling and provision during post-pregnancy care, thus, serve as key strategies to reduce repeat unintended pregnancies and to optimise maternal and child health. This holistic approach supports women in their reproductive life plan and reproductive autonomy, and can remove barriers to accessing chosen contraception. This is particularly important in the context of the increasing global restrictions around access to safe abortion care. This review article discusses the evidence and practical considerations for contraception delivery in the context of abortion, childbirth and early pregnancy loss (including miscarriage, ectopic pregnancy and gestational trophoblastic disease (GTD)).

### Contraception after abortion

Post-abortion contraception is a critical component of reproductive healthcare that prevents unintended pregnancies, reduces the need for subsequent abortions and supports women’s autonomy over their fertility (Schmidt-Hansen *et al.* 2020, Kim *et al*. 2021). Return of fertility can occur much earlier than expected following abortion and before the first post-treatment menstruation. One study reported the earliest ovulation 8 days after early medical abortion (although the average was around 3 weeks) (Schreiber *et al.* 2011). This emphasises the need for timely contraceptive counselling and provision. Integrating contraception into abortion care is an evidence-based strategy that significantly decreases the risk of subsequent unintended pregnancy. Previous research has also demonstrated that women are often motivated to discuss their future contraceptive needs at this time (Kavanaugh *et al.* 2011).

The World Health Organization’s (WHO) Medical Eligibility Criteria (MEC) for contraceptive use indicate that nearly all contraceptive methods can be safely initiated immediately after a first- or second-trimester abortion – except in rare cases of sepsis or complicated abortion (World Health Organization 2025). Delayed initiation is only recommended when there are medical contraindications, uterine infection or patient preference. There are, however, some specific considerations regarding contraception initiation at the time of abortion.

Co-administration of progestogen-based contraception alongside mifepristone (an anti-progesterone drug) does not appear to significantly reduce the efficacy of either drug (Hognert *et al.* 2016, Raymond *et al.* 2016*a*), except for the depot medroxyprogesterone acetate (DMPA) injection. In a randomised trial comparing same-day administration of intramuscular DMPA at the time of mifepristone versus delayed initiation post-abortion, there was a very small increase in the risk of ongoing pregnancy (3.6 vs 0.9% respectively) but no difference in rates of surgical intervention (Raymond *et al.* 2016*a*). Interestingly, patient satisfaction rates were also higher in the immediate initiation group, indicating that while the small difference in abortion failure rate is an important counselling point, it should not prevent immediate DMPA provision in patients who request this.

Oral contraceptives can be easily supplied at the time of abortion. The progestogen-only pill (POP) has very few contraindications for use and is safe for most people. Newer POPs containing desogestrel or drospirenone have improved side effect profiles and longer windows of administration than ‘traditional’ pills containing norethisterone (12 h for desogestrel, 24 h for drospirenone), and can reliably inhibit ovulation as effectively as the combined oral contraceptive pill. In some countries, the POP can be supplied from a diverse range of healthcare settings, including pharmacies, further removing barriers to access.

Where long-acting reversible contraceptives (LARC), such as the implant or intrauterine device (IUD), can be provided at the time of abortion care, this has been shown to more effectively meet the needs of women by reducing the rate of a future unintended pregnancy ending in abortion, compared to the provision of short-acting reversible contraception across diverse settings (Heikinheimo *et al.* 2008, Cameron *et al.* 2012, Rose & Lawton 2012, Kilander *et al.* 2016, Raymond *et al.* 2016*b*, Schmidt-Hansen *et al.* 2020). Randomised controlled trials comparing quick-starting the implant at the time of intake of mifepristone versus delaying insertion until the next period found that the former was associated with higher acceptability and fewer subsequent unintended pregnancies (Sonalkar *et al.* 2013, Hognert *et al.* 2016). Studies have also shown that providing an IUD immediately after first-trimester medical abortion is safe and leads to higher uptake and continuation than when insertion is delayed (Schmidt-Hansen *et al.* 2020).

However, the increase in telemedicine models for abortion care delivery can reduce in-person opportunities to provide women with their chosen contraception (Dixon *et al.* 2022). Contraception counselling remains an important feature within telemedicine consultations and short-acting methods, such as pills, can be easily supplied alongside abortion medication packs. However, since LARC methods require a trained provider to insert, their immediate initiation may not be possible. It is, therefore, crucial that rapid access pathways exist for women wishing to initiate a LARC method to do so as soon as possible. If LARC cannot be provided immediately, women can be offered a temporary ‘bridging’ method, such as the POP, if they wish. For individuals who are familiar with self-injection and who wish to use the progestogen-only injectable post-abortion, it should also be possible to provide supplies of subcutaneous medroxyprogesterone acetate for self-administration within telemedicine models of abortion care.

Effective post-abortion contraception depends on comprehensive counselling, method availability and respect for patient autonomy. The integration of newer approaches, such as audio-visual animation (Reynolds-Wright *et al.* 2020) and structured contraceptive counselling focusing on women’s desire to avoid pregnancy and effectiveness of available contraceptive methods, has been demonstrated as a useful tool to enhance discussion and to increase the uptake and continuation of contraception methods post-abortion (Emtell Iwarsson *et al.* 2021, Bizjak *et al.* 2024). Structured contraception counselling has also demonstrated high satisfaction amongst service users and providers (Envall *et al.* 2021) also in migrant populations (Iwarsson *et al.* 2022). A recent pilot showed that a post-telemedicine abortion follow-up text with the offer to discuss contraception and facilitate the chosen method of post-abortion contraception may be a useful intervention to help women who struggle to access their chosen method to initiate it (Reynolds-Wright & Cameron 2025).

In summary, contraception advice and provision following abortion is a cornerstone of reproductive healthcare. Immediate initiation of an effective method, tailored to individual medical eligibility and preferences, can prevent rapid repeat pregnancy and empower women to have control over their future reproductive health.

### Contraception after childbirth

The need for and provision of contraception after childbirth is less well established than in the post-abortion setting. The World Health Organisation recommends a minimum of 24 months spacing between birth and subsequent conception to optimise maternal and neonatal outcomes in future pregnancy (World Health Organization 2006). In some settings, a short inter-pregnancy interval of less than this is linked to an increase in the risk of preterm birth, low birth weight, stillbirth and an overall increase in neonatal mortality (Conde-Agudelo *et al.* 2006). Previous UK research has shown that 97% women do not plan to have another pregnancy shortly after childbirth (Heller *et al.* 2016). However, a study from the same part of the UK found that at least one in 13 women conceived again within a year and, amongst women attending for abortion care, a similar number had conceived within 12 months of a previous birth (Heller *et al.* 2016). In a Swedish retrospective cohort study, 2.5% of women had an abortion within 24 months after delivery (Lichtenstein Liljeblad *et al.* 2020).

In part, this may be linked to under-estimation of pregnancy risk after birth. Fertility can return quickly after birth, with the earliest ovulation reported around day 25 in non-breastfeeding women, with most ovulating by 6 weeks (Jackson & Glasier 2011). Evidence suggests that around 50% of individuals will also have resumed sexual activity by this time (McDonald & Brown 2013), leaving a window for unintended pregnancy unless contraception is initiated early. Women may also have misconceptions about the safety of certain contraceptive methods during the postpartum period and while breastfeeding (Thwaites *et al.* 2019), further limiting their contraceptive choices.

However, perhaps more significant are the challenges women can face trying to access effective contraception in the weeks after childbirth. In many countries, contraception has often been addressed as part of a routine postpartum follow-up visit a few weeks after birth. However, evidence indicates that these visits are often poorly attended and, increasingly in publicly funded settings, they may not be universally available (Lunniss *et al.* 2016, Perkins *et al.* 2024). Furthermore, there may not be adequate time to fully address the woman’s contraceptive needs, and additional appointments may be required to initiate a LARC method (Lunniss *et al.* 2016, Perkins *et al.* 2024). However, attending appointments in the weeks after birth to discuss or initiate contraception is difficult for women who are also balancing the needs of a newborn and perhaps other children in the household, while recovering from the birth itself. It is perhaps not surprising then that up to 50% of individuals do not attend booked follow-up appointments to initiate a LARC method, even when they have previously expressed a desire for this (Ogburn *et al.* 2005).

Integrating contraception into maternity care provides a unique opportunity to address this unmet need, as women are already in contact with the health system for delivery and newborn care. Although contraception is not strictly required before day 21 postpartum (when the earliest ovulation is reported), earlier or immediate postpartum initiation of most methods is safe and may be more convenient for women. However, introducing the subject of contraception for the first time on a postnatal ward also has its challenges. Both women and midwives report that a lack of privacy and time, and pre-occupation with the newborn, can limit the feasibility of this approach (Glasier *et al.* 1996, McCance & Cameron 2014).

Introducing contraception discussion earlier in the pregnancy journey, during the antenatal period, can offer several advantages. Previous research has shown that women value the opportunity to discuss contraception at this time (Freeman-Spratt *et al.* 2023). One study from the UK reported very high acceptability amongst women receiving a midwife-led contraception discussion during their 22-week antenatal visit (Cameron *et al.* 2017). There is often more time to discuss contraception and signpost to additional information to aid decision-making, allowing women the opportunity to fully consider their options. This relies on maternity staff having the knowledge, confidence and time to provide these, all of which have been identified as potential barriers (Botfield *et al.* 2021, Freeman-Spratt *et al.* 2023). Consequently, this is not yet a routine part of maternity care in most countries. Antenatal contraceptive counselling is also a pre-requisite to be able to provide contraception at the time of birth such as placement of an IUD during a caesarean section. A recent survey of experts from across Europe found that antenatal contraceptive discussion was routinely provided in less than half of the 28 countries surveyed (Cooper *et al.* 2024).

Several strategies are reported in the literature to support antenatal contraception information provision, including the use of digital health tools. In one pilot randomised controlled trial (RCT), a digital health intervention comprising a short audio-visual animation shown in the second trimester along with subsequent text message alerts during the remainder of pregnancy, demonstrated high satisfaction scores and knowledge recall amongst pregnant women when compared to in-person consultation alone (Cooper *et al.* 2025). However, to be truly effective, antenatal contraception information should ideally be partnered with rapid access to the full range of methods for women to start immediately after childbirth if they wish.

The WHO guidance supports the initiation of most methods immediately postpartum in otherwise medically eligible individuals (World Health Organization 2025). The exception to this is combined hormonal contraception, which should not be started prior to 3 weeks postpartum due to the risk of venous thromboembolism (VTE). For breastfeeding women (or those with additional risk factors for VTE), it should be delayed until at least 6 weeks. This guidance is due to limited and conflicting evidence about the effect of oestrogen-containing contraceptives while breastfeeding on infant outcomes (Tepper *et al.* 2016). However, progestogen-based methods are considered safe to use at this time, including by breastfeeding women, since no detrimental effects on lactation or infant development have been demonstrated (Phillips *et al.* 2016). Table 1 summarises the WHO method-specific guidance for initiation in the postpartum period (World Health Organization 2025).

**Table 1:** Adapted from World Health Organization’s Medical Eligibility Criteria (MEC)^†^ for contraceptive use, fifth edition 2015.

Condition	Cu-IUD	LNG-IUS	IMP	DMPA	POP	CHC
Postpartum breastfeeding	See below	See below				
a) 0 to <6 weeks			2	3	2	4
b) >6 weeks to <6 months			1	1	1	3
c) >6 months			1	1	1	2
Postpartum non-breastfeeding	See below	See below				
a) <21 days						
With other risk factors for VTE			1	1	1	4
Without other risk factors			1	1	1	3
b) >21–42 days						
With other risk factors for VTE			1	1	1	3
Without other risk factors			1	1	1	2
c) >42 days			1	1	1	1
Postpartum breastfeeding/non-breastfeeding			See above			
a) 0 to <48 h		1	1 or 2*			
b) 48 h to <4 weeks		3	3			
c) >4 weeks		1	1			
d) Postpartum sepsis		4	4			

* Not breastfeeding = 1; breastfeeding = 2.

^†^ The definition of the MEC categories is as follows – 1: A condition where there is no restriction for the use of method; 2: A condition where advantages of using method generally outweigh the risks; 3: A condition where risks generally outweigh advantages of using the method. Provision requires expert clinical judgement and/or specialist referral; 4: A condition which represents unacceptable health risk if the method is used.

Cu-IUD, copper intrauterine device; LNG-IUS, levonorgestrel intrauterine system; IMP, progestogen-only implant; DMPA, depot medroxyprogesterone acetate (progestogen-only injectable); POP, progestogen-only pill; CHC, combined hormonal contraception; VTE, venous thromboembolism.

Immediate postpartum provision of LARC methods (implants and IUDs) is associated with higher uptake and continuation compared to delayed initiation (Sothornwit *et al.* 2017), by removing barriers for women wishing to use these methods. However, the immediate postpartum provision of LARC depends on the availability of enough trained healthcare professionals in the maternity environment, which can be challenging to deliver. An observational study from US hospitals providing LARC methods from the maternity setting found an association with a reduction in short interpregnancy intervals and preterm birth rates (Steenland *et al.* 2022). There is evidence that immediate postpartum uptake of the implant amongst adolescent mothers is associated with fewer subsequent unintended pregnancies within the following year (Tocce *et al.* 2012, Han *et al.* 2014).

Immediate PPIUD insertion remains significantly under-utilised around the world, despite evidence supporting its safety and efficacy (Lopez *et al.* 2015, Sonalkar & Kapp 2015). While most of the early research was derived from low- and middle-income settings, in recent years, studies from the US, UK and other parts of Europe including Sweden, have further confirmed the feasibility and acceptability of this intervention (Heller *et al.* 2017, Cooper *et al.* 2020, Lichtenstein Liljeblad *et al.* 2022).

At caesarean birth, the IUD can be inserted through the uterine incision and placed at the top of the uterine cavity after removal of the placenta, with the threads directed towards the cervical canal. The threads often descend naturally into the vagina following resumption of menses. Currently available evidence suggests that this may be associated with an increased incidence of non-visible threads at a 6-week postnatal check (Heller *et al.* 2017). Therefore, access to ultrasound is an important part of the PPIUD aftercare pathway. PPIUD insertion at caesarean birth has been shown to be as safe as delayed insertion a few weeks later but is associated with higher IUD uptake and a lower rate of unintended pregnancy in the next year (Lopez *et al.* 2015, Sonalkar & Kapp 2015).

PPIUD insertion within the first 48 h after vaginal birth has also been shown to be safe, effective and highly acceptable (Cooper *et al.* 2020, Lichtenstein Liljeblad *et al.* 2022). Fundal placement can be achieved using a manual technique or, more commonly, using long placental (Kelly) forceps. The dilated cervix and ongoing effect of labour analgesia can make IUD insertion easier and less uncomfortable than a delayed insertion a few weeks later, with women reporting high satisfaction levels and lower-than-expected pain (Carr *et al.* 2018, Boydell *et al.* 2020). However, the dilated cervix and forces associated with postpartum uterine involution can lead to a higher rate of expulsion following IUD placement at this time.

The reported expulsion rate following vaginal PPIUD insertion varies considerably in the literature. A FIGO study of around 37,000 PPIUD insertions across six countries reported an overall expulsion rate of under 5% (Makins *et al.* 2018). However, clinician-led follow-up and ultrasound access were more limited than in high-income settings, where considerably higher expulsion rates have been observed in studies (Lopez *et al.* 2015, Sonalkar & Kapp 2015, Cooper *et al.* 2020). PPIUD expulsion rates also appear to be influenced by several factors including the timing of insertion, device type (hormonal or copper) and the experience of the provider (Averbach *et al.* 2020). Immediate post-insertion ultrasound has not been shown to reduce the likelihood of subsequent device expulsion (Dias *et al.* 2015) but can be useful to provide feedback about the accuracy of IUD placement during training and early service introduction, when expulsion rates are expected to be higher. The effect of cumulative provider experience was demonstrated in a recent study from the US, where a reduction in the service-wide PPIUD expulsion rate was observed from 28% in year 1 to 15% in year 3 (Free *et al.* 2024).

New innovations such as this could simplify the training and insertion process for PPIUD and reduce the impact of provider experience on expulsion rates in the future. A dedicated postpartum IUD inserter, which more closely resembles a standard IUD inserter, have been shown to perform as well as the forceps insertion technique but was favoured by providers (Blumenthal *et al.* 2018). However, despite these higher reported expulsion rates with PPIUD insertion, it remains a highly cost-effective intervention (Washington *et al.* 2015).

Contraception after childbirth is a cornerstone of reproductive and maternal healthcare. Immediate and effective postpartum contraception can reduce maternal and neonatal morbidity, promote healthy birth spacing and empower women to achieve their reproductive goals. Health systems should prioritise integrating contraceptive counselling and service delivery into all maternal care settings to ensure that every individual who has given birth is armed with the information and access they need to make informed contraceptive choices.

### Contraception after early pregnancy loss

Although contraceptive counselling is increasingly becoming part of maternity care, it is less well addressed at the time of early pregnancy loss. In one large UK population-based survey, two-thirds of respondents who reported experiencing miscarriage in the preceding year indicated a lack of pregnancy intention or ambivalence (Wellings *et al.* 2013) and in a Swedish setting 36.7% of those seeking emergency care in early pregnancy reported that the pregnancy was unintended (Bergman *et al.* 2022). Clinicians working in early pregnancy units report a level of discomfort in discussing contraception and may also lack the necessary knowledge to do so (Narayanan *et al.* 2022). However, fertility can return quickly after miscarriage, and while there is no medical need to delay further conception if this is desired, there is also a risk of future unintended pregnancy if not. Women can also experience similar barriers to accessing effective contraception as in other post-pregnancy situations.

The WHO indicates that all contraceptive methods are safe to start following an uncomplicated miscarriage, and this can be done immediately if desired. If hormonal methods are initiated more than 5 days after miscarriage management, the usual additional contraceptive cautions are needed until the method becomes effective. In the context of recurrent miscarriage (defined as three or more consecutive pregnancy losses), it is advisable to exclude underlying pro-thrombotic conditions (e.g. anti-phospholipid syndrome, thrombophilia) prior to use of oestrogen-containing contraception.

Most methods are also safe to initiate following an ectopic pregnancy regardless of how this is managed. Indeed, for women treated with methotrexate, conception should be avoided, and effective contraception is advised for 3 months after treatment due to its potential teratogenic effects (Elson *et al.* 2016). All contraceptive methods, except the lowest dose hormonal IUD, have been shown to protect against ectopic pregnancy. There can be provider misconceptions around the use of IUDs for individuals who have experienced ectopic pregnancy in the past. One Swedish study found that amongst women who experienced an ectopic pregnancy while using contraception, subsequent contraceptive uptake declined (particularly amongst IUD users), leaving them at increased risk of future pregnancy, including a further ectopic pregnancy (Nordström *et al.* 2020). It is true that there is a small increase in the relative risk of ectopic pregnancy in IUD users compared to non-users. However, as an IUD is one of the most effective methods of contraception, the absolute risk of any pregnancy (including an ectopic) occurring is very low, and indeed lower than less effective alternatives. The hormonal IUD with 52 mg levonorgestrel has been shown to provide a higher degree of protection from ectopic pregnancy compared to the lower-dosed hormonal IUDs in register-based studies and cohort studies (Elgemark *et al.* 2022). An IUD can be safely placed at the time of surgery for ectopic pregnancy and, thus, provide effective protection from new ectopic pregnancy.

A less common type of pregnancy loss, known as GTD, also presents important fertility and contraceptive considerations. GTD encapsulates a spectrum of disorders ranging from a partial or complete ‘molar’ pregnancy to rare malignant conditions such as invasive mole, choriocarcinoma and placental site tumours. In most cases, surgical evacuation of the uterus forms the mainstay of initial management, with subsequent disease monitoring through serum human chorionic gonadotrophin (hCG) levels. Since a further pregnancy during follow-up can negatively impact disease regression and prognosis, conception should be avoided. As such, contraception counselling (and ideally provision) is essential to successful GTD management.

Most recent guidelines now advise that any hormonal method of contraception can be safely started during management of GTD, including immediately after uterine evacuation and during hCG follow-up (Faculty of Sexual & Reproductive Healthcare 2017). However, IUD insertion should be delayed until serum hCG levels have returned to normal. This is due to concerns about the potential risk of uterine perforation or haemorrhage when an IUD is inserted into a large, vascular uterus.

Unfortunately, there have been relatively few innovations in the delivery of contraceptive care in early pregnancy settings, and further research is needed in this area.

## Conclusion

Post-pregnancy periods (postpartum, post-miscarriage and post-abortion) are high-leverage opportunities to provide effective contraception. Evidence and guideline consensus support offering a full range of methods, including same-day and immediate initiation of most options, with the clear benefits of increased uptake and prevention of rapid repeat pregnancy. Systems-level support (method availability, trained clinicians, appropriate follow-up pathways, and patient-centred information and counselling) is essential to translate evidence into improved outcomes for women and to support their reproductive autonomy.

### Future research

- Randomised controlled trials regarding the effectiveness in uptake and acceptability of antenatal contraceptive counselling.
- Randomised controlled trials comparing insertion techniques to reduce expulsion, particularly for immediate postpartum IUDs in vaginal delivery and at emergency caesarean sections.
- Interventions to facilitate access to LARC with telemedicine models of abortion care.
- Feasibility and acceptability of provision of contraception during management of early pregnancy loss.
- Implementation research exploring factors such as cost-effectiveness, training, and workflow models to overcome barriers to immediate post-pregnancy contraception provision.
- Interventions to tackle hesitancy around the use of hormonal contraception in post-pregnancy settings.

## Declaration of interest

HKK receives honorariums for lectures from AbbVie, Bayer, Gedeon Richter, Exeltis, Nordic Pharma, Natural Cycles, Mithra, Teva, Merck and Organon. HKK also provides expert opinion for Bayer, Gedeon Richter, Exeltis, Merck, Teva, TV4, Natural Cycles and Preglife. HKK is an investigator in trials sponsored by Bayer, MSD, Mithra, Gedeon Richter and Organon. HKK teaches in courses sponsored by Organon, Bayer and Gedeon Richter. HKK has written book chapters in *Guide for Contraception* (sponsored by Bayer) and in the Swedish medical products agency recommendation for contraception.

## Funding

This research did not receive any specific grant from any funding agency in the public, commercial or not-for-profit sector.

## Author contribution statement

MC and HKK were invited to prepare this review article. MC researched and prepared a first draft. Both authors reviewed the final manuscript prior to submission.
